# MRI in Chronic Pudendal Neuralgia: Diagnostic Criteria and Associated Pathologies

**DOI:** 10.3390/diagnostics16020326

**Published:** 2026-01-20

**Authors:** Michele Gaeta, Sofia Turturici, Karol Galletta, Carmelo Geremia, Attilio Tuscano, Aurelio Gaeta, Marco Cavallaro, Salvatore Silipigni, Francesca Granata

**Affiliations:** 1 Radiology Unit, Biomorf Department, University of Messina, Via Consolare Valeria 1, 98125 Messina, Italy; salvo.sili@gmail.com; 2 Neuroradiology Unit, Biomorf Department, University of Messina, Via Consolare Valeria 1, 98125 Messina, Italy; karolgall@yahoo.it (K.G.); marco88cavallaro@gmail.com (M.C.); fgranata@unime.it (F.G.); 3 Centro Pelvi Perineale UCP, Via Placida 12, 98121 Messina, Italy; dott.carmelogeremia@libero.it; 4 Gynecology Unit, Azienda Ospedaliera “Bianchi-Melacrino-Morelli”, Via Giuseppe Melacrino 21, 89124 Reggio Calabria, Italy; attiliotuscano@gmail.com; 5 Medicine and Surgery, University of Messina, Piazza Pugliatti 1, 98122 Messina, Italy; aureliogaeta15102004@gmail.com

**Keywords:** pudendal neuralgia, MRI, neuropathy

## Abstract

**Background/Objectives:** Chronic pudendal neuralgia is a relatively rare condition in the general population, with an incidence of 1%. Although diagnosis of pudendal neuralgia is mainly clinical, Magnetic Resonance Imaging (MRI) is commonly performed to obtain further information. However, clear criteria and guidelines for MRI diagnosis and the clinical–radiological correlation are still not definite. **Methods**: We reviewed 81 patients with chronic pudendal neuralgia, studied by an MRI designed protocol for a pelvis and pelvic floor examination. A key element of the protocol was the use of a diffusion-weighted imaging (DWI) technique with echo planar imaging (EPI) sequence (b-values of 0, 100, and 600) for the neurographic evaluation of the nerve. **Results:** MRI examination revealed DWI abnormalities in 42/81 patients. Pudendal nerve abnormalities were unilateral in 33/42 patients and bilateral in 9/42. Moreover, in 23/42 patients, pathologies related to a high probability of neuropathy have been identified. **Conclusions**: This study highlights the role of pelvic MRI as a valuable imaging modality in the evaluation of patients with chronic pudendal neuralgia. In the study protocol we propose, an essential role is played by the DWI technique, which improves the visual definition of the pudendal nerve and related anatomical structures. By focusing on anatomical visualization and structured image interpretation, our work provides a practical imaging-oriented contribution to a field in which standardized MRI evaluation is still lacking.

## 1. Introduction

**Figure 1:**
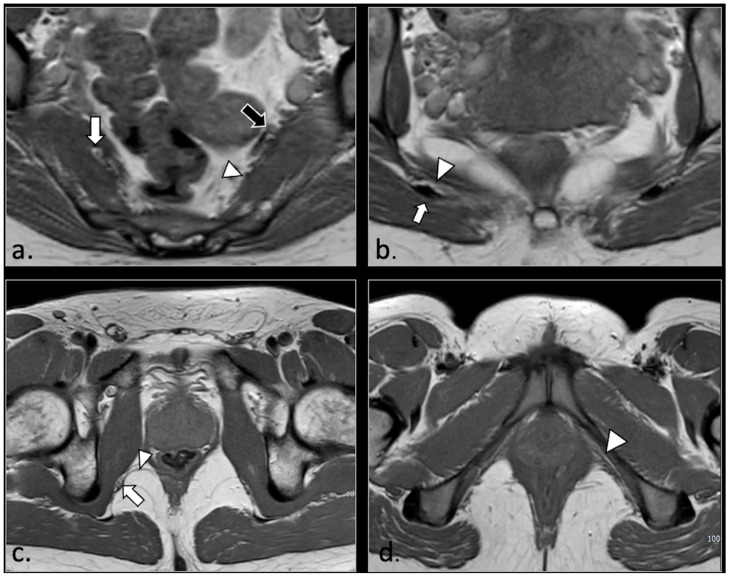
Representation of the anatomic course of pudendal nerve at four fundamental levels. (**a**) Axial T1-weighted scan shows trough piriform muscles and lumbosacral (black arrow) and pudendal (arrowhead) plexuses of the anterior surface of left piriform muscle. On the right side, the S2 spinal root runs into the piriform muscle (arrow). This anatomic variation can predispose to nerve entrapment and pudendalgia. (**b**) Axial T1-weighted scan at the extra-pelvic course of the pudendal nerve. The nerve runs in the space between sacro-tuberous ligament posteriorly (arrow) and coccygeal muscle and sacro-spinous ligament (arrowhead) anteriorly. (**c**) Axial T1-weighted scan at the level of proximal Alcock’s canal. The pudendal neurovascular bundle runs close to the obturator internal muscle (arrow). The transverse course of inferior rectal neurovascular bundle can be seen into ischiorectal fossa (arrowhead). (**d**) Axial T1-weighted scan at distal Alcock’s canal. At this level, it is difficult to detect the distal branches (arrowhead) of the pudendal nerve.

The pudendal nerve is a mixed sensory and motor nerve that supplies the perineum and external genitalia in both sexes. The nerve arises in the pelvic cavity from the anterior rami of spinal nerves S2, S3, and S4. The anatomical physiology of the nerve on MRI can be seen in Figure 1.

Pudendal neuralgia, caused by lesions of the pudendal nerve, is a chronic and severely disabling neuropathic pain syndrome affecting the sensory distribution of the pudendal nerve. Imaging techniques are increasingly used to investigate the anatomical substrates and potential sites of nerve involvement in this condition. It is a relatively rare condition in the general population, with an estimated incidence of 1 per 100,000, as reported by the International Pudendal Neuropathy Foundation [1]. However, its actual prevalence is likely underestimated and may affect up to 1% of the population, with women more than twice as commonly affected as men [2]. This syndrome is most commonly associated with the compression or entrapment of the pudendal nerve by posterior pelvic ligaments, particularly within the interligamentous space between the sacrospinous and sacrotuberous ligaments or within Alcock’s canal [3,4,5]. Nevertheless, a wide range of intra- and extra-pelvic pathological conditions may affect the pudendal nerve along its course, underscoring the need for imaging techniques capable of comprehensive anatomical assessment.

For the anatomical orientation, pudendal nerve evaluation can be structured by distinguishing intra-pelvic and extra-pelvic compartments. The intra-pelvic compartment includes the sacral nerve roots (S2–S4), sacral plexus, piriformis muscle, sacrospinous and sacrotuberous ligaments, ischial spine, obturator internus muscle, and pelvic sidewall. The extra-pelvic compartment comprises the lesser sciatic foramen, Alcock’s canal, ischioanal fossa, perineal soft tissues, and the terminal branches of the pudendal nerve. This anatomical framework underlies the etiological classification summarized in Table 1 and supports the interpretation of the imaging findings [2,3,4,5,6,7,8,9].

The diagnosis of pudendal neuralgia is primarily clinical, according to the Nantes criteria [3,4,5,10]. The symptomatology affects the perineal and genital regions in both sexes following a dermatomeric distribution [11]. The pain is usually worsened by sitting and relieved by standing or lying. Characteristically, patients are not awakened during sleeping. In addition, pain is not associated with objective sensory impairment and is relieved by a pudendal nerve block.

Pudendal neuralgia is frequently underdiagnosed, confused with other causes of chronic pelvic and perineal pain and inappropriately treated, causing a delay in proper management and severely negatively impacting the quality of life [3,10].

Imaging is essential to explore the potential causes of chronic pelvic pain and is decisive when it demonstrates a lesion able to account for the pudendal neuralgia [3].

Magnetic Resonance Imaging (MRI) obtained with 1.5- or 3-Tesla scanners provides a high-quality anatomical evaluation of the pelvic region, potentially allowing both the detection of intrinsic pudendal nerve neuropathy and the identification of extrinsic causes of nerve injury. Although 3-Tesla MRI is generally considered advantageous for peripheral nerve imaging due to its higher signal-to-noise ratio, optimized 1.5-Tesla protocols may still provide clinically meaningful anatomical information, particularly in routine clinical settings. Despite this, only a limited number of MRI studies focusing specifically on the pudendal nerve and syndromes related to its damage have been reported in the literature [12,13,14,15,16,17,18]. The aim of this study is to evaluate cases of pudendal neuralgia studied by MRI at our institution, to assess the site and mechanism of nerve damage, and to identify typical MRI diagnostic patterns.

## 2. Materials and Methods

We retrospectively analyzed 81 patients (53 females and 28 males) with pudendal neuralgia, aged 19 to 59 years. Demographic characteristics of the study population are summarized in Table 2.

Data collection spanned from 2017 to the present.

Patients with endometriosis, pelvic cancer, acute infections (e.g., perianal fistulas), and previous pelvic surgery were excluded.

Patients included in this study presented with a clinical picture strongly suggestive of pudendal neuralgia. Pudendal nerve block was not performed as part of the institutional diagnostic pathway. Patient selection was based on clinical evaluation supported by MRI findings consistent with pudendal nerve involvement.

In all patients with neuralgia in the territory of pudendal nerve innervation, we adopted an MRI protocol for pelvis and pelvic floor examination, using a 1.5 T MRI scanner (Ingenia, Philips Medical Systems, Den Haag, The Netherlands; software version 11.1 (2024-06-28)).

In all patients, MRI protocol included the following non-contrast enhanced sequences (total acquisition time about 20 min) for anatomic evaluation of pelvic region:

• Non-fat saturated axial, coronal, and sagittal T2-weighted fast spin-echo (FSE): TR 4000 ms, TE eff 100 ms, turbo factor 13, thickness 3 mm, parallel imaging SENSE AF 1.5.
• Fat saturated axial T2-weighted FSE: TR 4500 ms, TE eff 100 ms, turbo factor 13, thickness 3 mm. TR 4000 ms, ET eff 100 ms, turbo factor 13, thickness 3 mm, parallel imaging SENSE AF 1.5, fat saturation obtained by spectral pre-saturation with inversion recovery (SPIR) strong.
• Non-fat saturated Axial T1-weighted FSE: TR 450–550 ms, TE eff 10–15, turbo factor 3–5, thickness: 3 mm, parallel imaging SENSE AF 1.5.
• Coronal and sagittal SSTSE T2-weighted myelography for detection of Tarlov cyst.

In addition, in each patient EPI diffusion sequence encompassing all the course of the pudendal nerves, from sacrum to lower perineum, was acquired for neurography.

The parameters of the sequence were as follows: TR 4100 ms, TE 85 ms, 3b (0, 100, 600), acquisition matrix 125 × 100, EPI factor 41, compressed sense acceleration with reduction factor of 2.5, fat-suppression obtained by SPIR. The sensitivity of DWI-based techniques is related both to the intrinsic characteristics of peripheral nerves, which are composed of diamagnetic molecules, and to their histological organization, which results in anisotropic diffusion of water along nerve fibers [19,20].

## 3. Results

In 42 patients, we observed hyperintensity of the pudendal nerve on DWI-weighted MR scans. Pudendal nerve abnormalities were unilateral in 33/42 patients and bilateral in 9/42.

In 23/42 patients, pudendal nerve abnormalities are found in association with extra-neural abnormalities which with high probability played a role in the onset of pudendal neuralgia. Specifically, we observed the following pathologies:

(1) Hemorrhagic Tarlov’s cyst of the sacrum (1 patient);
(2) Unilateral or bilateral hypertrophy of the pyriform muscle (4 patients);
(3) Unilateral or bilateral lesions of the sacrotuberous and/or sacrospinous ligaments (interligamentous space) (5 patients);
(4) Unilateral rupture of puborectal and/or pubococcygeal muscle (4 patients);
(5) Perineal fibrosis involving Alcok’s canal (4 patients);
(6) Giant cyst of the prostatic utricle (1 patient);
(7) Pudendal nerve schwannomas (2 patients);
(8) Varices of the pudendal vein in the Alcock canals (2 patients).

**Figure 2:**
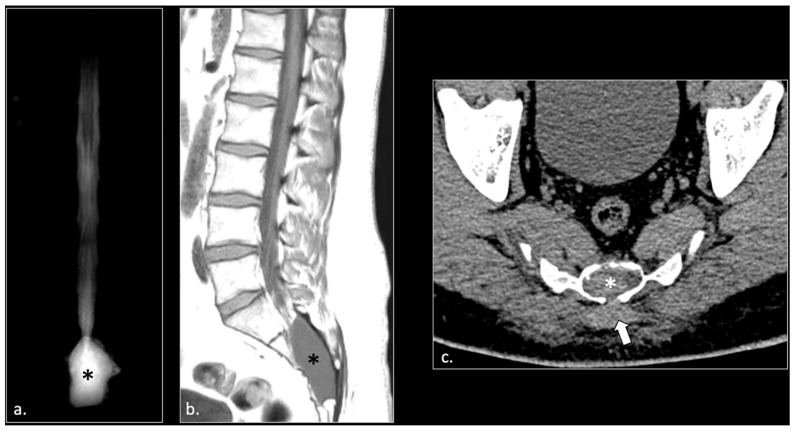
A 24-year-old male with chronic pudendalgia. The onset of pudendalgia could be referred to a sacral trauma. (**a**) Coronal MRI myelography shows a large Tarlov cyst in the sacral canal (*). (**b**) Sagittal T1 scan of the lumbar spine demonstrates slight hyperintensity of the cyst, due to chronic hemorrhage (*). (**c**) Computed tomography (CT scan) obtained 15 months before MRI examination, at the time of acute trauma, shows acute hemorrhage into the cyst (*) and a Morel Lavallée hematoma (arrow) in the deep subcutaneous tissue of the sacral region. Acute fracture of the sacrum around the cyst could be seen with a bone window (unshown).

**Figure 3:**
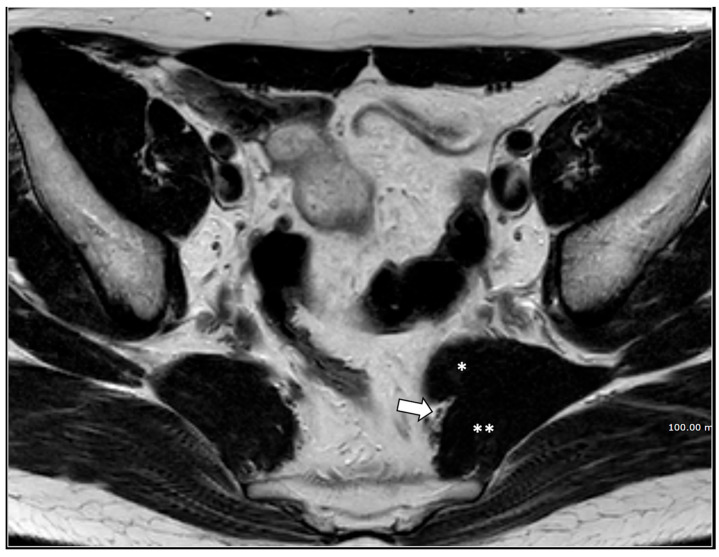
A 37-year-old male with pudendal neuralgia. Axial T2-weighted scan through mid-pelvis shows hypertrophy of the left piriformis muscle. Roots of the lumbosacral plexus lie on the anterior surface of the muscle. On the other hand, S2 and S3 roots of the pudendal plexus (arrow) can be seen along the medial surface of the muscle within an incomplete canal created by the two bellies (* and **) of the muscle.

**Figure 4:**
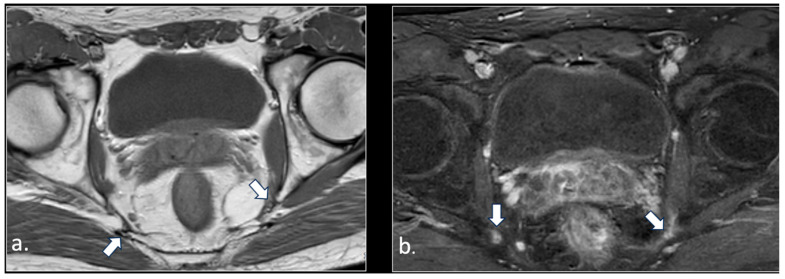
A 32-year-old man with relapsing pudendalgia after decompressive surgery on both pudendal nerves. (**a**) Axial T1-weighted image, obtained at the level of the ischial spine, shows fibrosis along the extra-pelvic course of both pudendal nerves (arrows). (**b**) Axial T1-weighted enhanced subtracted scan demonstrates enhancement along the course of the nerves (arrows).

**Figure 5:**
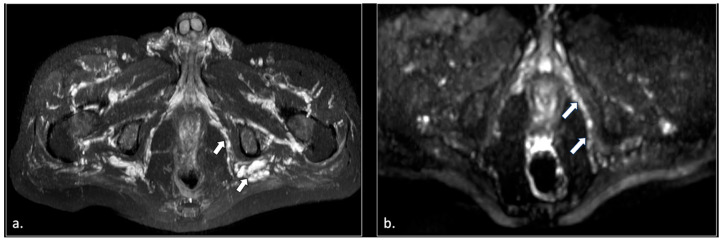
A 71-year-old male with chronic pudendalgia. (**a**) STIR maximum intensity projection (MIP) scan shows diffuse venous dilatation of the lower pelvis, including varices of pudendal veins (arrow). (**b**) b600 DWI maximum intensity projection (MIP) shows enlargement and hyperintensity of the left pudendal nerve (arrow).

**Figure 6:**
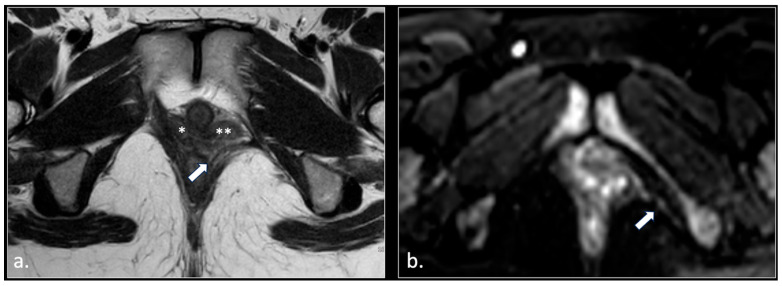
A 43-year-old female with chronic post-traumatic pudendalgia. (**a**) Axial T2-weighted scan shows a normal right side pubo-coccygeus muscle (*). On the left side, an avulsion of the pubo-coccygeus muscle can be seen. The residual left pubo-coccygeus muscle is thinned (arrow). The vagina is shifted on the left side (**). (**b**) Axial b600 DWI scan demonstrates diffuse hyperintensity of the left pudendal nerve (arrow).

**Figure 7:**
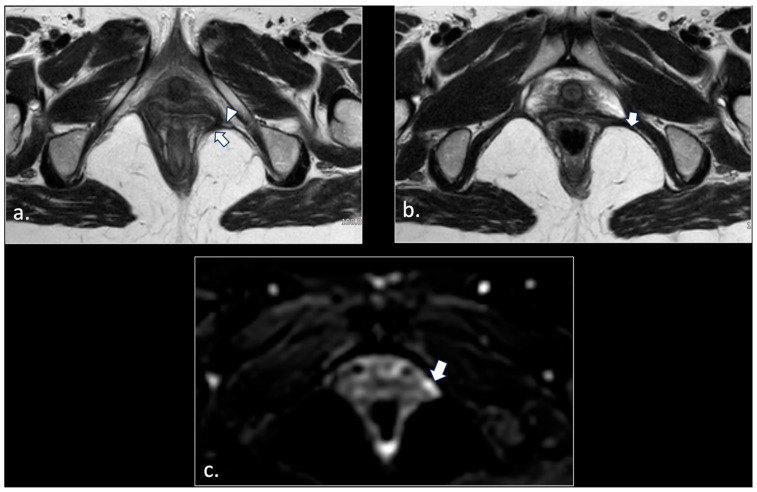
(**a**) Axial T2-weigthed section through the distal Alcock’s canal shows an atrophy of the left pubo-coccigeal muscle (arrow) and paravaginal fibrosis on the same side. (**b**) A more cranial section shows with better advantage the paravaginal fibrosis (arrow); (**a**) the wall of the vagina is stretched (arrowhead). (**c**) b600 DWI section, acquired at the same level of (**b**), shows a strong hyperintensity and enlargement of the left pudendal nerve (arrow).

**Figure 8:**
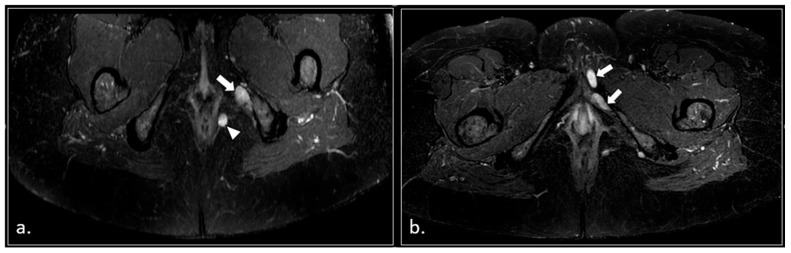
A 50-year-old female with chronic pudendalgia. (**a**) STIR maximum intensity projection (MIP) image demonstrates schwannomas of the left pudendal nerve (arrow) and inferior rectal nerve (arrowhead). (**b**) A lower section shows two schwannomas of the distal branches of the left pudendal nerve (arrow).

Some of these cases are shown (Figure 2, Figure 3, Figure 4, Figure 5, Figure 6, Figure 7 and Figure 8).

**Figure 9:**
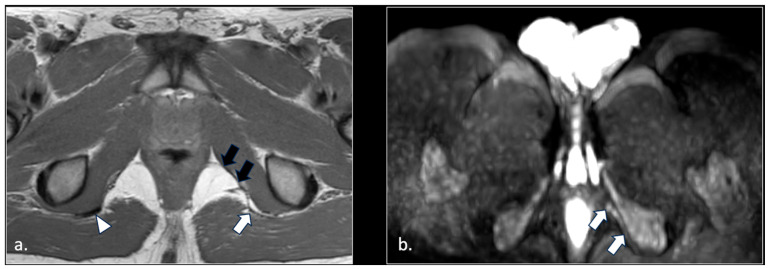
A 33-year-old man suffered from disabling perineal and scrotal pain lasting 3 years. The patient had been an amateur cyclist active for about 10 years. Palpation along the course of the left pudendal nerve, at the Alcock canal and medial to the ischial spine, reproduced the patient’s pain, consistent with the Valleix phenomenon [21]. MRI showed in an axial T1-weighted scan (**a**) a thinning of the left sacrotuberous ligament (white arrow), likely due to chronic microtrauma from cycling. Note the normal right sacro-tuberous ligament (arrowhead). Moreover, a thickening of the medial wall of the left Alcolck’s canal can be seen (double black arrows). The left pudendal nerve in the Alcock canal was hyperintense (arrows) on the b600 diffusion-weighted scan (**b**). A block of the left pudendal nerve was performed, with temporary relief (1 week) of symptoms.

Rapresentative Case (Figure 9).

## 4. Discussion

The pudendal nerve is a mixed sensory, motor, and autonomic nerve arising from the anterior rami of the second, third, and fourth sacral nerve roots (S2–S4) [4,22,23]. Within the pelvic cavity, it courses along the anterior surface of the piriformis muscle and exits the pelvis through the lower part of the greater sciatic foramen, passing between the piriformis and coccygeus muscles. The nerve then follows a short extra-pelvic course within the interligamentous space between the sacrospinous and sacrotuberous ligaments, curving around the ischial spine before re-entering the pelvis through the lesser sciatic foramen. Distally, it runs forward within the pudendal (Alcock’s) canal, together with the internal pudendal vessels, along the lateral wall of the ischiorectal fossa, where it is enclosed by a sheath of the obturator fascia [4,22,23]. Along this pathway, particularly at the level of the sacrospinous ligament and within Alcock’s canal, the pudendal nerve gives rise to its terminal branches, including the inferior rectal nerve and the dorsal nerve of the penis or clitoris, and represents a critical anatomical site predisposed to mechanical irritation or entrapment. Pudendal neuralgia is an uncommon condition in the general population, with an estimated prevalence of approximately 1%, and occurs more frequently in women [1]. The condition affects the genital and perineal regions in both sexes and is characteristically worsened by sitting.

According to Nantes criteria [3], the key clinical points for diagnosis are represented by the following:

(1) The topographical distribution of the pain (from the anus to the penis or clitoris);
(2) Exacerbation of pain when sitting;
(3) Lack of awakening during the night;
(4) Lack of objective sensory alterations;
(5) Response to pudendal nerve block.

Complementary diagnostic features include burning, shooting, or stabbing pain; numbness; allodynia or hyperpathia; a sensation of a rectal or vaginal foreign body; progressive worsening of pain during the day; predominantly unilateral symptoms; pain triggered by defecation; and dyspareunia.

Pudendal neuralgia is most commonly related to the compression or entrapment of the pudendal nerve along its anatomical course. A structured classification based on intra- and extra-pelvic compartments, together with the distinction between primary and secondary etiologies, has been widely adopted for the evaluation of deep gluteal space pathologies and has been applied in the present study to the specific context of pudendal neuralgia. This framework provides a practical and reproducible approach for multidisciplinary clinical assessment. Accordingly, the relevant anatomical structures have been outlined, and the intra- and extra-pelvic causes of pudendal nerve involvement are summarized in Table 1, facilitating the interpretation of imaging findings.

Furthermore, increasing evidence indicates that anatomical variability within neural plexuses is common, including variations involving the S3 nerve root and its relationships with adjacent nerves [11]. Such variability may influence symptom presentation and contribute to nerve entrapment syndromes, supporting the need for a comprehensive diagnostic approach that integrates detailed clinical evaluation with targeted MRI within an intra- and extra-pelvic assessment framework. In fact, MRI provides good anatomical definition of the pelvic region, with visualization of the pudendal nerve, especially in its proximal segments. For this purpose, high spatial resolution (3 mm thick) T1- and T2-weighted scans along three Euclidean planes and 4 mm-thick EPI diffusion with b600 are particularly suitable (Figure 1). MRI assessment of the distal branches of the pudendal nerve becomes more difficult, even after surgical marking [13].

T2-weighted fat-suppressed and STIR sequences represent the most commonly used and effective MRI techniques for performing MR neurography and for detecting peripheral nerve diseases. However, these sequences are frequently affected by signal interference caused by blood flow within adjacent vessels, resulting in vascular hyperintensity that may hinder the evaluation of both normal and pathological nerves and constitute a potential source of diagnostic pitfalls [24,25]. Diffusion-weight imaging (DWI), acting as a “black-blood” sequence, overcomes this limitation by facilitating the distinction between neural and vascular structures. Cardiac-triggered T2-weighted fat-suppressed sequences have been proposed to address this issue [15,16]; however, this approach is time-consuming and does not consistently achieve sufficient suppression of the vascular signal. Similarly, 3D T2-weighted fat-suppressed and fast-STIR neurography performed after intravenous administration of gadolinium chelates has been shown to improve the neurography quality of the lumbar and brachial plexuses by enhancing vascular signal suppression. Nevertheless, suppression of the signal from small peripheral vessels remains imperfect, and the use of gadolinium increases the examination time and cost, while also exposing patients to potential adverse reactions [26,27]. In the present study, this limitation was addressed through the use of diffusion-weighted imaging with a b value of 600, which is not affected by vascular hyperintensity. Under normal conditions, lumbar and sacral nerve roots appear hyperintense on DWI after exiting the neural foramina, and the sciatic nerve typically demonstrates hyperintensity along its entire course. In contrast, the normal pudendal nerve does not show increased signal intensity and is generally not visible on b600 DWI images.

Image evaluation was performed by two radiologists with 30 and 10 years of experience, respectively, and the final assessment was reached by consensus.

The most common cause of pudendal neuralgia is considered to be entrapment between the posterior pelvic ligaments (the sacrotuberous and sacrospinous ligaments) compressing or trapping the pudendal nerve within the interligamentous region and/or at its entrance to the Alcock’s canal [1,2,3,4,22,23].

In addition, the pudendal nerve can be involved at the level of the sub-piriform canal, resulting in the piriform syndrome, which can cause both sciatica and, less frequently, pudendalgia. Entrapment of the distal branches of the pudendal nerve (dorsal nerve of the clitoris/penis) can occur at the level of the distal Alcock’s canal.

The onset of pudendal neuralgia is often related to difficulties during childbirth (secondary to excessive stretching), trauma, sequelae of surgeries and radiotherapy, prolonged bicycle riding, pelvic fractures, and neoplasms.

Tarlov cysts have been reported in the literature as another potential cause of chronic lumbosacral and pelvic pain. Specifically, they are often located in the distribution of the pudendal nerve origin at the sacral nerve roots S2, S3, and S4, and it has been postulated that they may cause symptoms like pudendal neuralgia [28,29]. However, a recent study questions the actual role of Tarlov cysts in the genesis of pudendal neuralgia [29]. In our cohort, a case of Tarlov cyst involved in the traumatic fracture of the sacrum was considered as the probable cause of pudendal neuralgia since surgical decompression of the cyst relieved the symptoms.

Only a few previous studies on the pudendal nerve and the diagnostic potential of MRI in patients with pudendal neuralgia have been reported in the literature [12,13,14,15,16,17]. Most of these focus on MRI neurography of the pudendal nerve, by emphasizing that the hyperintensity of the nerve is a crucial element for the diagnosis of neuropathy.

Magnetic resonance neurography is based on sequences that suppress background tissues (including signal from small vessels) and highlight water content within nerves.

With the recent advancement in MR hardware and imaging techniques, several approaches to the MRI neurography of peripheral nerves have been proposed [30,31,32,33,34,35,36,37,38,39,40,41].

In our experience, unlike other districts and for reasons related to the anatomical peculiarities of the pudendal nerve, vessels’ hyperintensity on T2-weighted fat-sat and STIR sequences, used to obtain MRI neurography, makes the diagnosis of pudendal neuropathy quite challenging. In addition, different MRI techniques were used and reported in the radiological literature and the MRI diagnostic criteria for pudendal neuropathy were not clearly defined. In approximately half of our patients, we identified a DWI signal alteration of the pudendal nerve using thin-slice axial scans with an appropriate b value.

**Figure 10:**
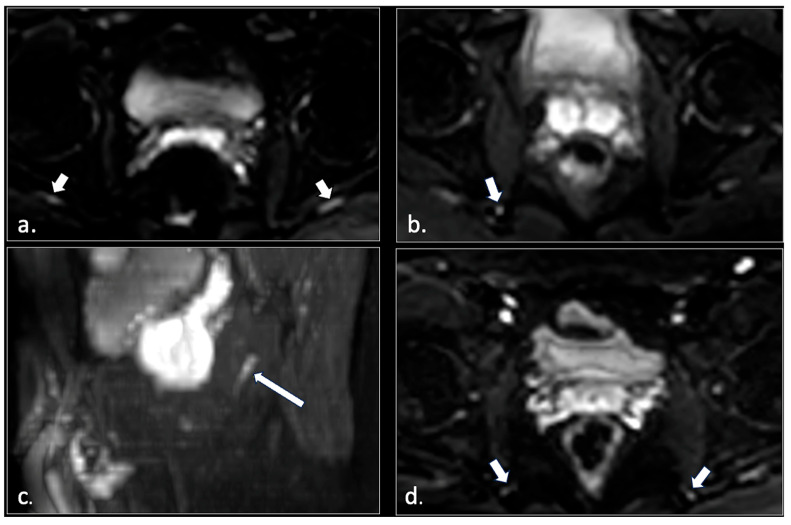
In Figure 10 are shown images related to diffusion-weighted imaging (DWI) scans. (**a**) does not show abnormalities of pudendal nerve. Note the physiological hyperintensity of the sciatic nerve (arrows). (**b**) depicts hyperintensity of the right pudendal nerve at the level of ischial spine. In the same patient, a sagittal reconstruction (long arrow in **c**) shows hyperintensity of the right pudendal nerve along the proximal Alcock’s canal. In (**d**) a case of bilateral hyperintensity of the nerve can be seen.

Therefore, based on these observations, we propose simple but effective diagnostic MRI criteria for assessing pudendal neuropathy. Specifically, the pudendal nerve should be considered abnormal if there is evident hyperintensity (comparable to that of the normal ipsilateral sciatic nerve) along its course on two consecutive or three non-consecutive b600 DWI images (Figure 10).

Finally, it is necessary to emphasize that in our cohort 39 out of 81 patients with symptoms compatible with pudendal neuralgia had no abnormalities on MRI examination. The cause of this negativity on MRI is not known, and the topic is worthy of further study. In this context, pelvic MRI findings should be interpreted within the broader framework of the hip–spine–pelvis complex, as disorders involving adjacent anatomical or biomechanical regions may contribute to symptoms despite unremarkable pelvic imaging. Probably, some of these cases are due to acquired or genetically related (e.g., Charcot-Marie-Tooth) sensory neuropathies of the peripheral nerves [42], not detectable with an MRI examination due to the limits of spatial and contrast resolution.

### Limitations

Our paper suffers from three main limitations:

1. The absence of a correlation with the pudendal nerve block test. All patients included in this study presented with a strong clinical and anamnestic suspicion of pudendal neuralgia and were referred for pelvic MRI as part of their diagnostic pathway. Assessment of the diagnostic performance of MRI compared with pudendal nerve block as a reference standard was beyond the scope of the present work and would require dedicated future studies. Nevertheless, pelvic MRI performed using a dedicated imaging protocol, as in our study, may potentially contribute to the diagnostic process, support clinical decision-making, and be helpful in selected cases in which the nerve block results are inconclusive or the procedure is difficult to perform. Furthermore, MRI may aid in identifying the underlying etiology of pudendal neuralgia.
2. All patients were studied with a 1.5 Tesla scanner. Since a 3 Tesla scanner improves the spatial resolution [13], we could expect better results with such a type of technology signal. Further studies performed with a 3 Tesla scanner on groups of patients with pudendal neuralgia should be obtained.
3. We did not explore the potential role of new techniques such as 3D T2-weighted sequence with blood signal suppression by a pre-saturation pulse, which is a promising technique for studying small nerve branches without interference from small vessel signaling [38,39,40,41]. To the best of our knowledge, reports on the use of this approach in patients with pudendal neuralgia have not yet been published. However, there are some clear advantages in our approach, based on diffusion neurography in patients with pudendal neuralgia. These advantages are:
  (1) The technique is available on every 1.5 and 3 Tesla scanners;
  (2) It is solid and reliable in absence of metallic orthopedic hardware;
  (3) The administration of gadolinium is not required.

## 5. Conclusions

In our study, we focused on the application of MRI in patients with a clinical diagnosis of pudendal neuralgia. Pelvic MRI represents a valuable imaging modality that provides detailed anatomical information and plays an important role in the evaluation of patients with suspected pudendal nerve involvement. We also proposed an MRI protocol and diagnostic considerations emphasizing the role of diffusion-weighted imaging (DWI), a rapid, robust, and widely available technique that enhances visualization of the pudendal nerve and related anatomical structures and contributes significantly to imaging-based assessment of pudendal neuralgia.

## Figures and Tables

**Table 1:** Intra-pelvic and extra-pelvic causes of pudendal neuralgia.

Location	Etiological Category	Causes	Pathophysiological Mechanism
Intra-pelvic	Anatomical entrapment	Entrapment within Alcock’s canal; compression between sacrospinous and sacrotuberous ligaments; anatomical nerve variants	Chronic mechanical compression
	Muscular and myofascial	Obturator internus hypertonicity; levator ani spasm; pelvic floor myofascial pain	Dynamic compression, ischemia, neurogenic inflammation
	Gynecological	Deep infiltrating endometriosis; post-surgical adhesions; large uterine fibroids	Infiltration, traction, or extrinsic compression
	Urological	Chronic prostatitis	Inflammatory neuropathy
	Colorectal	Chronic proctitis; pelvic abscesses or fistulas	Direct nerve irritation or fibrosis
	Vascular	Pelvic venous congestion; pelvic varices	Pulsatile or static vascular compression
	Neoplastic	Pelvic tumors (rectal, prostate, gynecological)	Direct infiltration or mass effect
	Iatrogenic	Gynecological, urological, and colorectal surgery; mesh implantation; post-prostatectomy changes	Direct nerve injury, fibrosis, or entrapment
Extra-pelvic	Mechanical compression	Entrapment at the lesser sciatic foramen; distal perineal branch compression	Distal nerve entrapment
	Postural, functional, and sport-related	Prolonged sitting; sedentary lifestyle; lumbopelvic imbalance; cycling; horseback riding; sports with pelvic overload	Repetitive microtrauma; chronic compression or vibration-induced neuropathy
	Traumatic	Pelvic trauma; ischiopubic fractures; perineal falls	Direct nerve damage
	Obstetric	Prolonged vaginal delivery; instrumental delivery	Nerve stretches and ischemic injury
	Extra-pelvic surgical	Anal or perineal surgery; hemorrhoidectomy; episiotomy	Partial nerve injury or post-surgical fibrosis
	Infectious/dermatological	Sacral herpes zoster; deep perineal infections	Inflammatory neuropathy
	Radicular/central	Lumbosacral radiculopathy (S2–S4); sacral canal stenosis	Referred neuropathic pain
	Central sensitization	Chronic pelvic pain syndrome	Amplification of nociceptive signaling

**Table 2:** Demographic characteristics of the study population.

Variable	Value
Number of patients	81
Female sex, *n* (%)	53 (65.4%)
Male sex, *n* (%)	28 (34.6%)
Age range (years)	19–59

## Data Availability

The original contributions presented in this study are included in the article. Further inquiries can be directed to the corresponding authors.
